# Far-Red Absorbing Rhodopsins, Insights From Heterodimeric Rhodopsin-Cyclases

**DOI:** 10.3389/fmolb.2021.806922

**Published:** 2022-01-21

**Authors:** Matthias Broser

**Affiliations:** Institute for Biology, Experimental Biophysics, Humboldt-Universität zu Berlin, Berlin, Germany

**Keywords:** NIR-absorption, retinal-chromophore, color-tuning, microbial rhodopsin, heterodimeric rhodopsin-cyclase, fluorescent protein

## Abstract

The recently discovered Rhodopsin-cyclases from Chytridiomycota fungi show completely unexpected properties for microbial rhodopsins. These photoreceptors function exclusively as heterodimers, with the two subunits that have very different retinal chromophores. Among them is the bimodal photoswitchable Neorhodopsin (NeoR), which exhibits a near-infrared absorbing, highly fluorescent state. These are features that have never been described for any retinal photoreceptor. Here these properties are discussed in the context of color-tuning approaches of retinal chromophores, which have been extensively studied since the discovery of the first microbial rhodopsin, bacteriorhodopsin, in 1971 (Oesterhelt et al., Nature New Biology, 1971, 233 (39), 149–152). Further a brief review about the concept of heterodimerization is given, which is widely present in class III cyclases but is unknown for rhodopsins. NIR-sensitive retinal chromophores have greatly expanded our understanding of the spectral range of natural retinal photoreceptors and provide a novel perspective for the development of optogenetic tools.

## Introduction

**FIGURE 1:**
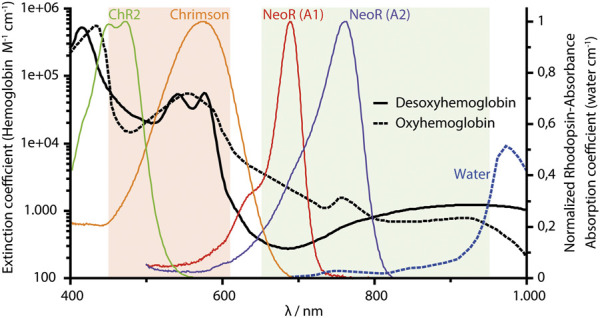
NIR-window of biological tissue (650–950 nm, indicated as olive area). The spectral range (450–610 nm) of microbial rhodopsin shown orange, and absorption spectra of two ChR (ChR2 and Chrimson) commonly used in optogenetics. NeoR absorption (red absorbing-state) with either A1-retinal or A2-(3,4 Dihydro-) retinal. All rhodopsin spectra are normalized to their maximum retinal absorbance. Chrimson spectrum from J. Vierock (Humboldt University Berlin), spectra of hemoglobin and water obtained from S. Prahl, 1998 (scott.prahl@oit.edu), using data from W.B. Gratzer, Medical Research Council Labs, Holly Hill, London, United Kingdom and N. Kollias, Wellman Laboratories, Harvard Medical School, Boston, MA, United States. https://omlc.org/spectra/hemoglobin/summary.html.

Microbial rhodopsins have become highly attractive research tools, since these photoreceptors allow the manipulation of cellular functions by light (Deisseroth, 2015). While the most widely utilized rhodopsin in optogenetics is still Channelrhodopsin (ChR), which enables neuroscientists to activate or inhibit neuronal transmission, the growing family of newly discovered microbial rhodopsins, aided by the availability of genomic data, has provided a treasure trove of novel photoactivated proteins with diverse functions (Govorunova et al., 2017). An evolving application of microbial rhodopsins is their use as membrane voltage sensors that provide an optical readout of neuronal activity. This approach is based on the observation that in some proton-pumping rhodopsins, the fluorescence of retinal is modulated by the membrane potential (Kralj et al., 2012). The success of rhodopsins as optogenetic tools is due to the fact that their endogenous *all-trans-*retinal chromophore is present in sufficient quantities in most cellular tissues, including the mammalian brain. However, light penetration of biological tissue is severely limited by hemoglobin absorption and scattering, leaving a spectral window of highest transparency in the near-infrared (NIR) range between 650 and 950 nm (Figure 1). Since microbial rhodopsins hardly absorb light in this spectral region, optogenetic experiments on living animals or possible therapeutic applications depend on implanting the stimulating light source close to the target cell population. Similarly, as rhodopsins feature broad absorption peaks, the spectral range for the simultaneous application of different optogenetic tools within multicolor approaches is limited due to pronounced cross-activation. To overcome this restriction, several experimental attempts have been made to shift the light sensitivity of rhodopsins into the NIR range. The first and still one of the most commonly used ChR is ChR2 from *Chlamydomonas reinhardtii* (Nagel et al., 2003), which has an absorption maximum in the blue (λ_MAX_ ∼470 nm, Figure 1) in comparison to the maximal sensitivity at 610 nm of Ruby-ACR, the most red-shifted light-gated ion channel from heterotrophic protist Labyrinthulea (Govorunova et al., 2020). This already remarkably broad spectral range of retinal photoreceptors (∼450–610 nm, Figure 1) allows the combined and simultaneous use of several optogenetic tools, e.g., for stimulation and inhibition of neuronal activity (Vierock et al., 2021). However, despite the intensive research that has been conducted on the color-shifting principles for retinal proteins since the discovery of bacteriorhodopsin (BR) as the first microbial rhodopsin almost 5 decades ago (Oesterhelt and Stoeckenius, 1971), the absolute limits of this chromophore's spectral tuning remain unknown.

### Enzymerhodopsins

Among microbial rhodopsins, Enzymerhodopsins represent a new functional and structural class in which the rhodopsin photosensor is directly fused to a cytoplasmic enzyme domain within one polypeptide chain. So far, three different types of Enzymerhodopsins have been reported: the Histidine kinase rhodopsins [HKR (Kateriya et al., 2004)], the Rhodopsin phosphodiesterases [RhoPDE (Yoshida et al., 2017)], and the Rhodopsin guanylyl-cyclases [RGC (Avelar et al., 2014), Figure 2A] and a detailed discussion of the optogenetic potential of current Enzymerhodopsins is given in (Mukherjee et al., 2019; Tsunoda et al., 2021). All currently characterized Enzymerhodopsins are involved in the regulation of the ubiquitous second messenger cGMP. Genome analysis of the green algae *C. reinhardtii* has predicted HKRs shortly after the discovery of ChR (Kateriya et al., 2004), but the functional characterization of these large and complex proteins that comprises several cytoplasmic modules that are known from the two-component signaling systems has been difficult. Hence, it has taken almost 15 years to characterize them as light-inhibiting guanylyl-cyclases that are triggered by a phosphorylation-cascade (Tian et al., 2018a). The sequence of RhoPDE from the choanoflagellate *Salpingoeca rosetta* has been derived from genomic data. Its initial functional characterization as light-activated phosphodiesterase was hampered by the high dark activity and the promiscuity with respect to the substrates cGMP and cAMP (Lamarche et al., 2017; Yoshida et al., 2017; Tian et al., 2018b). Recent crystal structures of its rhodopsin domain with and without the cytoplasmic linker has provided the first structural details of an Enzymerhodopsin (Ikuta et al., 2020). These structures allow for the placement of the additional N-terminal transmembrane helix found canonically for Enzymrhodopsins adjacent to TM2 and 3 at the outer face of the dimer among other things. Recently, the function of RhoPDE was assigned to light-driven collective contractility in the choanoflagellate *Chanoeca flexa,* inverting the curvature of multicellular cup-shaped colonies (Brunet et al., 2019). Although the protein sequences of RGC became available after those of HKR or RhoPDE, it was the first Enzymerhodopsin with proven functionality. This photoreceptor type was discovered in the aquatic fungus *Blastocladiella emersonii* in 2014 and has been linked to the phototaxis of fungal zoospores (Avelar et al., 2014). Only 1 year later, two groups independently characterized the light-activated guanylyl cyclase activity and proved its aptitude for optogenetic applications (Gao et al., 2015; Scheib et al., 2015). Among the Enzymerhodopsins characterized so far, RGCs show the lowest dark-activity and the fastest photocycle kinetics (in the ms time regime), which are both suitable properties to enable tight light-controlled cGMP production. Since the isolated class III cyclase of RGC is constitutively active, the rhodopsin keeps the enzyme in an inactive conformation in the dark, while light-activation of the photosensor propagates to the active site and enables its catalytic capabilities (Scheib et al., 2018). Despite their different functionalities, all Enzymerhodopsins listed above have several features in common: 1) they comprise eight transmembrane α helices with both termini located cytoplasmatic, 2) they function as homodimers, and 3) their maximal spectral sensitivity is between blue (∼490–527 nm for RhoPDE) and green (∼530–550 nm for RGC and HKR). However, the number of characterized proteins of each photoreceptor type [with three HKRs (Luck et al., 2012; Tian et al., 2018a), nine RhoPDEs (Sugiura et al., 2020), and five homodimeric RGCs (Gao et al., 2015; Scheib et al., 2015)] is still small, and the spectral range may increase in the future. For HKR1 from *C. reinhardtii,* the rhodopsin domain was found to exhibit bistable photochemistry, which allows switching between a blue absorbing (480 nm) and UV-absorbing (380 nm) state (Luck et al., 2012). However, photoactivated enzyme function was only shown in artificial chimera constructs with HKR2 (Tian et al., 2021). Nevertheless, this indicates that there is more variation regarding photochemical properties for HKRs.

**FIGURE 2:**
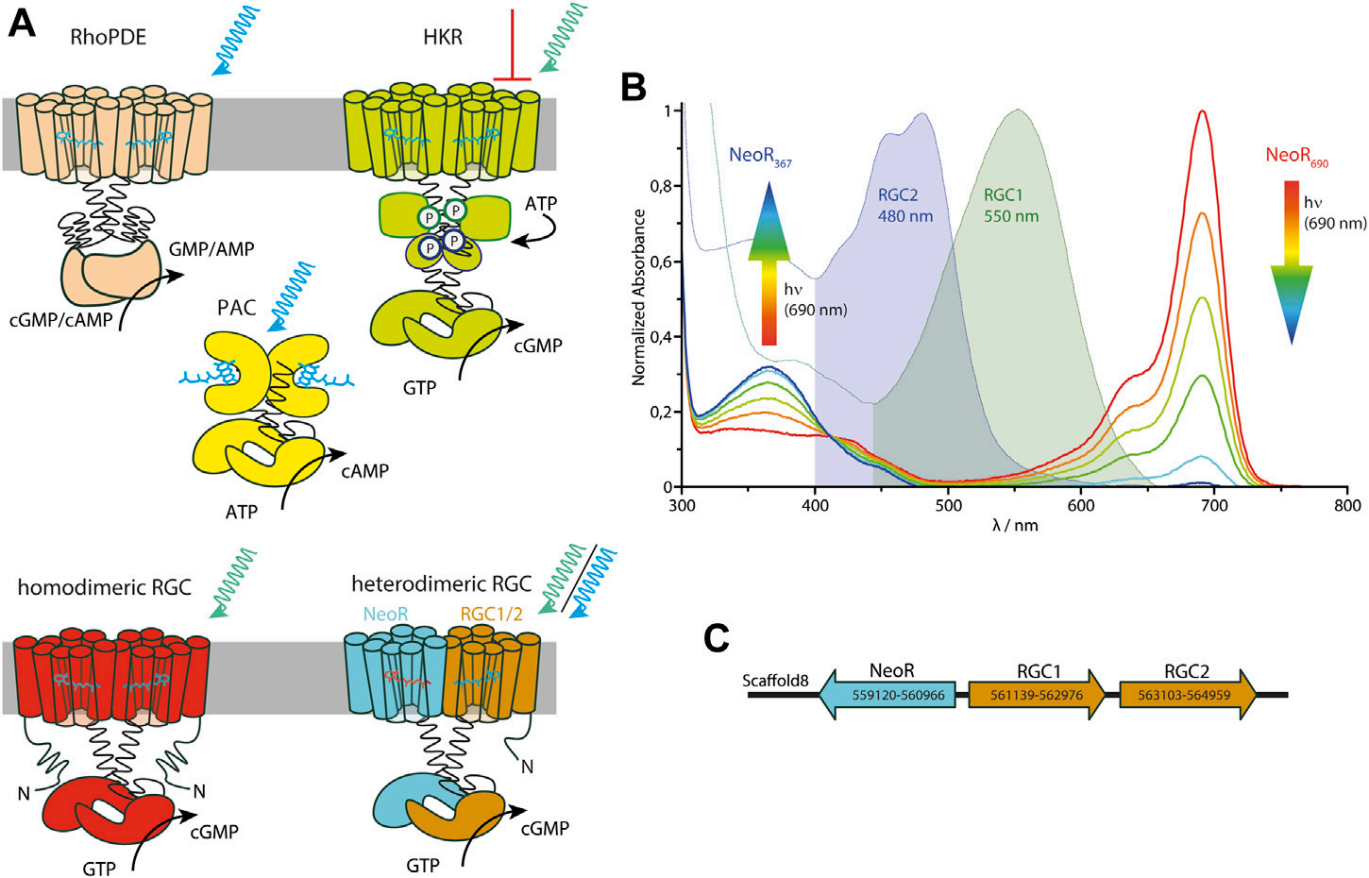
**(A)** Cartoon of all three types of currently known Enzymerhodopsins: Rhodopsin-Phosphodiesterase (RhoPDE), Histidine-Kinase Rhodopsin (HKR) and homodimeric and heterodimeric Rhodopsin-guanylyl Cyclases (RGC), complemented by soluble photoactivated adenylyl-cyclase (PAC) from bacteria containing a BLUF-photosensor (Stierl et al., 2011). Blue/green-light triggered enzyme activation is indicated by arrows (for HKR green-light inhibits cyclase function). **(B)** Spectra of the three RGCs—RGC1, RGC2, and NeoR—from *R. globosum* with the two photoconvertable states of NeoR (λ_MAX_ given as indices). The rainbow-colored arrows symbolize the NIR radiation associated with the spectral progression of the bistable photoconversion. **(C)** Genomic arrangement of RGC-genes in *R. globosum*. Positions of start- and stop-codons on scaffold 8 from genomic sequencing are given by numbers (Mondo et al., 2017).

### Heterodimeric RGCs

Among the fungal kingdom, the two neighboring phyla Blastocladiomycota and Chytridiomycota are characterized by the production of motile zoospores. *Rhizoclosmatium globosum,* a species that belongs to the class of Chytridiomycetes, has three RGC genes—encoding for RGC1, RGC2, and Neorhodopsin (NeoR) (Broser et al., 2020). In contrast to other Enzymerhodopsins, these RGCs do not produce a functional photoreceptor when expressed individually. Instead, both RGC1 and RGC2 only form light-activated guanylyl-cyclases in conjunction with NeoR. This indicates that the functional photoreceptor-complex is a heterodimer consisting of RGC1/NeoR or RGC2/NeoR (Figure 2A). The two rhodopsin-domains of the heterodimeric complex show very different photochemical properties. While both RGC1 and 2 have conventional green or blue absorbing retinal chromophores, NeoR absorbs maximally in the near-infrared spectral region peaking at 690 nm (Figure 2B), which is by far the most red-shifted rhodopsin described until now (Broser et al., 2020). Action-spectroscopy using cGMP gated reporter-channels assigned the light-triggered enzyme activation of the heterodimeric photoreceptor to the green/blue absorbing chromophore of RGC1/2. The observed transient photocurrents suggest that both, RGC1 and 2, feature a similar photocycle to homodimeric RGCs (Scheib et al., 2015). Notably, NeoR is a bistable photochromic rhodopsin that, in analogy to HKR1 from *C. reinhardtii*, can be photoconverted between a far-red absorbing state (NeoR_690_) and an UV-absorbing state (NeoR_367_) with a deprotonated retinal Schiff base (Figure 2B). Although the mechanism remains unknown, photoconversion of NeoR_690_ to NeoR_367_ increases the photoresponse of NeoR/RGC2 complexes. This suggests that NeoR takes on a regulatory role (Broser et al., 2020). Thus, the three RGCs of *R. globosum* form a photosensory system that enables light perception in the entire visible spectral range, including UVA and NIR (Figure 2B). Interestingly, all three RGC genes—NeoR, RGC1, and RGC2—are clustered in the genome of *R. globosum* with RGC1 and 2 sequentially arranged as tandem while NeoR is coded by the complementary strand [as shown in Figure 2C (Mondo et al., 2017)]. Such an arrangement is known from other fungi genomes (e.g., yeast) and allows concerted bidirectional transcription of the respective genes from a single transcription start region (Xu et al., 2009). Therefore, some regulation of the heterodimeric RGC may already occur at the level of transcription.

In the NIR-absorbing state, NeoR_690_ is highly fluorescent with a fluorescence quantum yield of 20%. Both far-red absorption and high fluorescence are unusual properties for rhodopsins. Nevertheless, due to the impact for optogenetic applications, several attempts have been made to color-tune microbial rhodopsins toward this near infrared region or to increase the fluorescence quantum yield of those used as voltage-sensors. In this contribution, the strategies for designing the photochemical properties of retinal-chromophores are discussed in the context of the native NeoR system, the by far most red-shifted rhodopsin.

### Tuning the Spectral Properties of Retinal Chromophores

**FIGURE 3:**
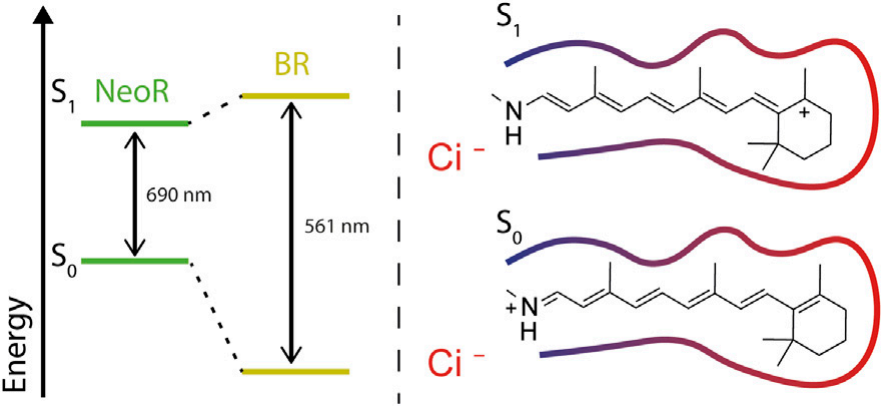
Energy diagram of electronic ground (S0) and first excited (S1)-state defining the wavelengths of the absorbed light; right: charge transfer of the RSBH^+^ in S1-positive charge locates at the β-ionone ring, where it is stabilized by polar surrounding (red contour-line). Negatively charged counterions (indicated as Ci^−^) stabilizing the positive charge of the RSBH^+^ in the ground state (S0).

The mechanism of how the absorption properties of the retinal chromophore is tuned by the protein’s surroundings has been the subject of intense research for microbial and animal rhodopsins. Early theoretical calculations on protonated retinal Schiff base (RSBH^+^) chromophores already revealed that the electronic character of the π-electron system significantly differs upon ejecting an electron from S0 to the first excited state S1. The positive charge in the ground-state (S0) is mainly localized at the RSBH^+^-nitrogen, whereas transition to the S1-state can lead to a transfer of positive charge toward the β-ionone ring (Warshel, 1978) (Figure 3). With this, the character of alternating single and double bonds [described as bond lengths alternation (BLA)] changes and even may become inverted, thereby facilitating the isomerization reaction. According to the different charge distribution, electrostatic interaction with the RSBH^+^-nitrogen tunes the energy level of S0 while the surrounding of the polyene, especially at the β-ionone ring, targets the S1 state (Figure 3). Indeed, this has led to the formulation of the so-called two-point charge model (Warshel, 1978; Nakanishi et al., 1980) in which two counterions stabilize the positive charge either at the RSBH^+^-nitrogen (S0) or at the β-ionone ring (S1). However, a strong ionic interaction with the β-ionone ring may not be compatible with retinal binding, since such a constellation was not observed in any rhodopsin. Historically, the absorption maximum at ∼440 nm of the (RSBH^+^) in organic solvents has been taken as a reference point to describe the protein-induced, bathochromic opsin-shift (Nakanishi et al., 1980). However, taking into account that the RSBH^+^ absorption measured *in vacuo* peaks at ∼610 nm (Andersen et al., 2005), one should consider the opsin-shift as a spectral blue-shift caused by a counterion complex that stabilizes the positive charge localized at the RSBH^+^-nitrogen, as this lowers the electronic ground state (S0) energy of the chromophore. In microbial rhodopsins, the counterion complex is built by conserved carboxylates positioned at helix 3 and 7. However, with the growing number of known rhodopsin sequences, it has become clear that the RSBH^+^ site arrangement is more diverse. HKR2, e.g., lacking any carboxylates at common counterion positions, still shows maximum sensitivity to green-light (Tian et al., 2018a). Therefore, other interactions likely involving water-molecules and ions may account for stabilization of the RSBH^+^. Intensive experiments mutating the retinal binding pocket of microbial rhodopsins, particular of BR, have been undertaken to prove this concept (Derguini et al., 1983; Ahl et al., 1988; Mogi et al., 1988; Mogi et al., 1989; Marti et al., 1991; Greenhalgh et al., 1992; Greenhalgh et al., 1993). However, the results were not always conclusive, which may reflect the problem that introduced point-mutations potentially alter the integrity of the retinal binding pocket more globally than intended. Nevertheless, the obtained data have been included in recent machine-learning approach, that provides a statistical model of the relationship between amino-acid residues and absorption properties (Karasuyama et al., 2018).

The highest spectral variance ranging from 340 to 700 nm has been proposed from action-spectroscopy of the compound eye of stromatopods [Mantis shrimps, (Marshall et al., 2007)]. However, the spectral sensitivity determined by electrophysiology on intact eyes may be affected by internal filtering systems, and a precise spectral description of the responsible NIR photoreceptor is still pending. Based on thermodynamic considerations, Barlow has proposed that shifting the absorption of any visual pigment toward lower energy is accompanied by increase noise that is produced by thermal isomerization (Barlow, 1957), thus resulting in lower light-sensitivity of the highly amplified visual systems in animals. Indeed QM/MM simulations on bovine rhodopsin have revealed that the (S0) transition state of thermal isomerization shares the charge-transfer structure of the excited state (S1) (Gozem et al., 2012). According to this, protein-chromophore-interactions that lower the energy gap between S0 and S1 will also reduce the energy barrier for thermal isomerization.

A detailed—but artificial—approach for investigating the color-tuning principles of retinal chromophores uses a mutant human retinol-transporting protein (hCRBPII) that binds all-trans retinal as RSBH^+^ via an introduced Lysine-residue (Wang et al., 2012). Mutating the residues of the retinal cavity results in chromophores with the most red-shifted absorption maximum at 644 nm, even red-shifted with respect to RBS^+^ *in vacuo*. These studies combine highly resolved x-ray crystallographic data and theoretical simulation to decipher the contributions of different structural regions of the retinal binding pocket on the observed opsin-shift. Their results have identified two main principles for the obtained red-shift: an equal distribution of polarizable residues along the polyene chain and a reduction of stabilizing interactions to the RSBH^+^-nitrogen, albeit this system lacks any counterion (Wang et al., 2012; Suomivuori et al., 2016). Notably, the crystal structures obtained for various hCRBPII mutants has shown only negligible differences regarding the planarity of the retinal, which confirms that steric constraints are not responsible for the color-shift (Wang et al., 2012). A similar conclusion has been depicted from the analysis of retinal chromophores derived from the structures of various microbial rhodopsins (Hayashi et al., 2001; Luecke et al., 2001; Hoffmann et al., 2006). Detailed theoretical investigations on protein-induced opsin-shifts became available with the crystal structure of sensory rhodopsin II [SRII (Luecke et al., 2001)], which shares a similar counterion-site and retinal geometry as BR but absorbs blue light (SRII λ_MAX_ 499 nm; BR λ_MAX_ 561 nm). Hybrid quantum mechanics/molecular mechanics (QM/MM) simulation has assigned the major influence for the blue-shifted absorption of SRII to the exchange of polar residues close to the β-ionone ring (Hoffmann et al., 2006).

More recent studies have utilized high throughput methods in order to determine the (multiple) amino-acid exchanges that alter the photochemical properties by directed evolution (McIsaac et al., 2014; Engqvist et al., 2015). This approach identifies variants with red-shifted λ_MAX_ up to 626 nm due to the substitution of two residues of the DTD/DTE motive conserved in proton-pumping rhodopsins. The observed spectral-shift is synergistic and cannot be derived additively from the individual mutants. Since one of the mutations targets the counterion and proton acceptor of the RSBH^+^, none of the proteins show light-driven proton transport. Thus, for ion-pumping rhodopsins, which require the functional integrity of the RSBH^+^ region for unidirectional transport, the natural ability to trim absorption by modulating the counterion-site appears to be limited. Nevertheless, the pumping is retained for a 40 nm red-shifted (λ_MAX_ 565 nm) sodium-pumping rhodopsin carrying two mutations that only moderately change the polarity close to the β-ionone ring and the RSBH^+^ without disturbing its functional arrangement (Inoue et al., 2019). With the availability of genomic data of various microbes, extensive screening of natural ChR sequences has been employed to identify color- or other variants with suitable properties for optogenetics (Klapoetke et al., 2014). This has led to the discovery of several ChR families with strikingly different structural and functional features regarding gating-mechanism, ion-selectivity, and photocurrent kinetic (Govorunova et al., 2021). The multiple mechanisms that have evolved for light-gated ion channels work to engage the residues of the RSBH^+^ region in different ways. This suggests that there is some flexibility regarding the arrangement of the counterion complex and allows for the development of broader spectral sensitivity. Accordingly, ChRs show an extraordinarily broad natural spectral variation ranging from an absorbance maximum at 445 nm [found for cation-conducting ChR from marine alga (Govorunova et al., 2013)] to 610 nm in the recently described anion-conducting ChRs [Ruby-ACR (Govorunova et al., 2020)]. Even the structurally more similar cation-conducting ChRs from the single genus Chlamydomonas contain red-absorbing Chrimson (λ_MAX_ 590 nm, *C. noctigama*) and blue-absorbing ChR2 (λ_MAX_ 470 nm, *C. reinhardtii*) and thus show a higher color variance than most ion-pumping rhodopsins (Hou et al., 2012). The Chrimson and ChR2 crystal structures indicate that the different protonation state of the counterions and an altered distribution of polar residues close to the β-ionone ring causes the spectral shift (Volkov et al., 2017; Oda et al., 2018). An artificial color-tuning strategy to blue shift the absorption introduces mutations that force the β-ionone ring towards a twisted 6-s-cis configuration as in visual rhodopsins (Kato et al., 2015). This prevents an efficient conjugation of the ring electrons and therefore increases the energy gap of the electronic transition with minor changes of the counterion site.

### NIR-Absorbing Neorhodopsin

**FIGURE 4:**
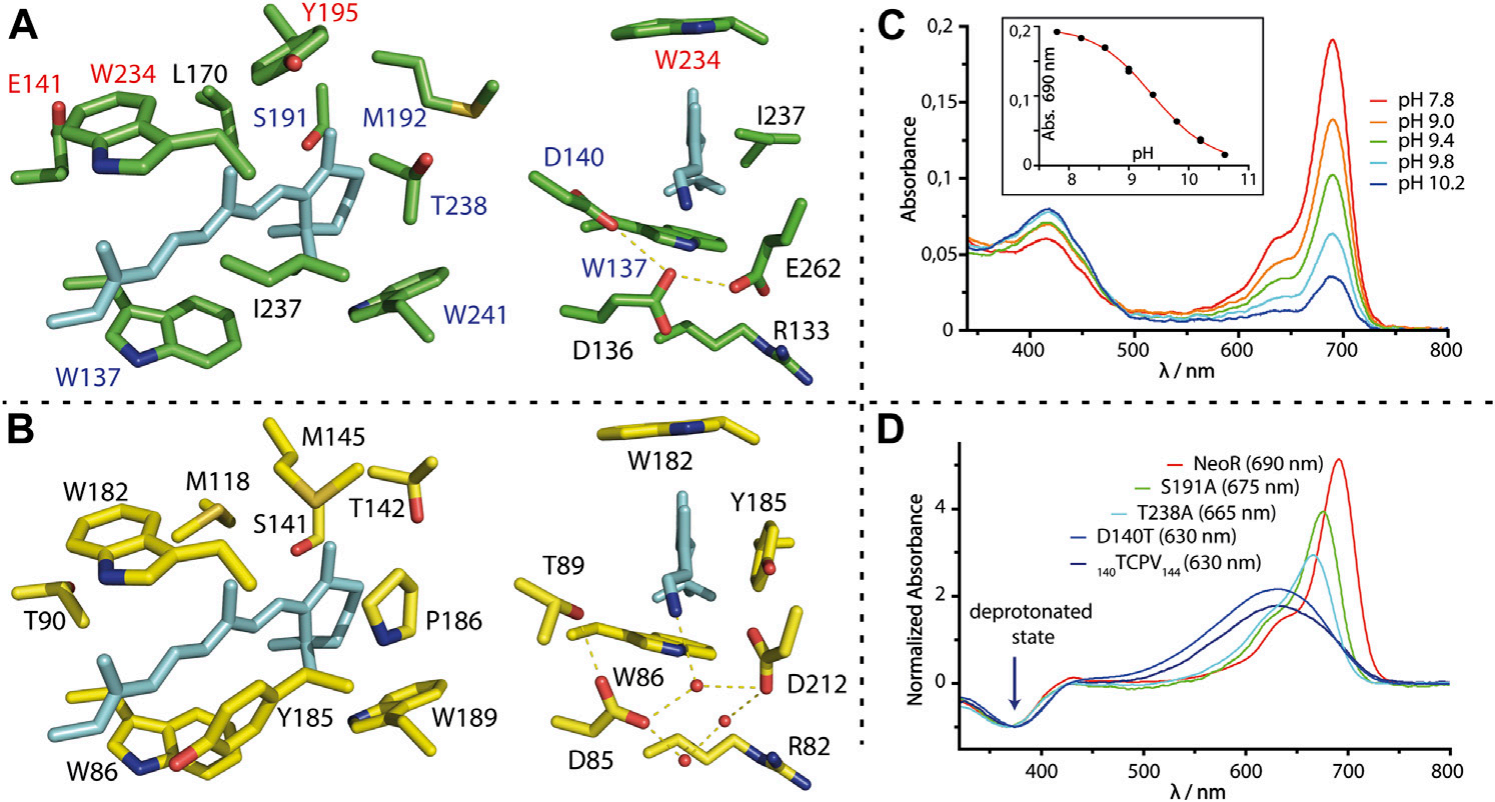
**(A,B)** Retinal binding cavity residues of NeoR (green) and BR [yellow, 2NTU (Lanyi and Schobert, 2007)]; NeoR homology model is based on the structure of RhoPDE [7CJ3 (Ikuta et al., 2020)]. Right: View towards the RSBH^+^ counterion-complex with water molecules (red spheres) and hydrogen bonds (dashed lines). **(C)** pH-titration of NeoR. Absorption at 690 nm irreversibly disappears at high pH, while a photochemical inactive form evolves with broad peak-absorption at ∼420 nm. Inset: titration curve with apparent pK_A_ of 9.4. **(D)** Absorption-difference spectra (red minus UV-state) of selected bistable NeoR mutants that shift absorbance to the blue. Spectra are normalized to the UV-(deprotonated-) state. _140_TCPV_143_: quadrupole mutant _140_DEAN_143_ to a TCPV motif commonly present in microbial rhodopsins.

Given the already broad spectral range of the many different natural rhodopsins, further extended by engineered mutants, the encounter of Neorhodopsin (NeoR) from *R. globosum* with an absorption spectrum peaking at 690 nm and thus red-shifted by 80 nm compared to any other native rhodopsin before has been unexpected. This extreme red-shift resembles ∼50% of the known absorption range of microbial rhodopsins (λ_MAX_ ∼ 450–610 nm, Figure 1 and Table 1) on the nm scale. Thus, NeoR significantly expands the spectral window of this photoreceptor class. Another untypical feature of the NeoR absorption is the narrow spectral bandwidth (NeoR: FWHM <900 cm^−1^) contrasting the broad and mostly unstructured spectra that is commonly observed for rhodopsins or polyenes. Indeed, similar narrow spectra have only been reported for BR reconstituted with non-natural retinal-analogs (Derguini et al., 1983). Despite the extraordinary red-shifted absorption, the NeoR retinal binding pocket reveals a surprisingly similar architecture to other microbial rhodopsins (Figure 4A). The presence of three carboxylates E136, D140, E262 at the RSBH^+^-site is unique for NeoR. Both glutamates occupy common counterion-positions found in the vast majority of microbial rhodopsins in which an aspartate usually occupies the position homolog to E262 (e.g., D212 in BR, Figure 4B). In most rhodopsins, threonine resides homolog to D140, e.g., as part of the DTD/DTE motif of various proton pumps, while aspartate serves as counterion at this position in sodium-pumping rhodopsin (Inoue et al., 2013). The finding that the by far most red-shifted microbial rhodopsin NeoR contains three potentially negative counterions is counterintuitive, as it suggests that the positive charge of the RSBH^+^-nitrogen can be effectively stabilized and thereby promotes blue-shifted absorption. Nevertheless, the arrangement of RSBH^+^ counterions in rhodopsins is structurally more complex and often involves water molecules and other interactions, which affects the proton affinity of carboxylates and thus the net charge. A prominent example is the red-absorbing ChR Chrimson that nominally is sharing the two carboxylate-residues with other chlorophyte ChRs. However, one of the counterions in Chrimson protonates at a neutral pH, which weakens the interaction with the RSBH^+^ in the red absorbing form. Deprotonation at alkalic conditions strongly blue-shifts the absorption towards the spectral range that has been observed for other chlorophyte ChRs (Oda et al., 2018). In analogy, bathochromic-shifted absorption is observed due to protonation of D85 in BR at low pH (Okumura et al., 2005).

**TABLE 1:** (Upper panel) Fluorescence properties of selected rhodopsin and phytochrome derived NIR fluorescent proteins. (Lower panel) Optical properties of natural red-absorbing microbial rhodopsins.

Fluorescent protein	λ_Max_ Excitation/Emission	QY/Brightness	References
NeoR	690 nm/707 nm	20%/25.8	Broser et al. (2020)
Archon 2^a^ ^,^ ^b^	586 nm/∼735 nm	1.05%/0.04	Piatkevich et al. (2018); Penzkofer et al. (2020)
QuasAR 1^a^ ^,^ ^b^	580 nm/∼740 nm	0.65%/-	Hochbaum et al. (2014)
Arch-7^a^	616 nm/727 nm	1.2%/1.26	McIsaac et al. (2014)
Mero-6^a^ ^,^ ^c^	759 nm/770 nm	13%/10.7	Herwig et al. (2017)
iRFPP713^d^	690 nm/713 nm	6%/6.3	Filonov et al. (2011)
miRFP670-2^d^	643 nm/670 nm	13.6%/14.0	Matlashov et al. (2020)

Rhodopsin	λ_Max_ Absorption	Function	References
RGC2/NeoR (Heterodimer)	480 nm*/690 nm	Rhodopsin-Cyclase	Broser et al. (2020)
	*light-activation at 480 nm		
Ruby-ACR (*Hf*ACR1)	610 nm^e^/595 nm	Anion-Channel	Govorunova et al. (2020)
Chrimson/ChrimsonSA^f^	590 nm/608 nm (SA-mutant)	Cation-Channel	Klapoetke et al. (2014); Oda et al. (2018)
*Hs*SR I	587 nm	Light-Sensor	Bogomolni and Spudich (1982)
*Hs*HR	575 nm	Chloride-Pump	Taylor et al. (1983)

a Archaerhodopsin-3 based fluorescent rhodopsins.

b Voltage-sensors.

c Containing non-native merocyanine-chromophore.

d Containing tetrapyrrole-chromophore (bilin) . Fluorescence quantum yield (QY [%]); Brightness [QY*extinction coefficient/1000].

e Derived from Photocurrent-Actionspectrum.

f Red-shifted Chrimson-mutant. *Hs*SR I, *Hs*HR Sensory-Rhodopsin 1, Halorhodopsin from *Halobacterium salinarium*.

QM/MM simulations of NeoR employing all possible protonation states of the three titratable groups does suggest that only either E136 or E262 is deprotonated (Broser et al., 2020). Notably, these two QM/MM optimized models show no direct contact between the negatively charged glutamate and the RSBH^+^-nitrogen, which instead potentially forms a hydrogen-bond to the protonated D140. The lower electrostatic stabilization of the RSBH^+^ in NeoR must allow for a substantially greater delocalization of the positive charge along the retinal in comparison to other rhodopsins, which causes the energy levels of ground state and excited state to converge (Figure 3). The pH-titration of NeoR shows that far-red absorption irreversibly disappears with an apparent pK_A_ of ∼9.4, while a photoinactive state is formed absorbing at ∼420 nm [Figure 4C, (Broser et al., 2020)]. This indicates that the RSBH^+^ region of NeoR is prone to structural alterations induced by deprotonation. In line with this result, mutations of E136 or E262 to glutamine have resulted in deprotonated and photochemical inactive chromophores and replacement with the shorter aspartate abolished retinal binding at all (Broser et al., 2020). The contribution of D140 is less clear. According to the QM/MM simulations, D140 is protonated, a conclusion that has been supported by the D140N mutant, which retains the spectral properties of NeoR. Replacing the residue with a polarizable cysteine (D140C, λ_MAX_ 676 nm) moderately blue-shifts the absorption, while a substantial shift (λ_MAX_ 630 nm) and spectral broadening is obtained for D140T mutant (Figure 4D). This is the residue present in most microbial rhodopsins. These findings could point towards a higher ground-state at this position dynamic after the intrusion of water to the RSBH^+^ that has been induced by the mutation. Indeed, molecular dynamic simulations suggest that water is effectively shielded from the RSBH^+^ region in wildtype NeoR, which presumably favors both far-red absorption and narrow spectral shape. Still, NeoR D140T reveals a bistable retinal-chromophore, indicating that the primary photochemistry is retained in the mutant. Similar spectral properties have been obtained for a NeoR quadruple mutant in which the residues 140–143 mimics a common motif found in various rhodopsins and indicates only a minor influence of the amino acids following D140 (Figure 4D).

The contribution of the other regions of the putative NeoR retinal binding pocket on the spectral properties has been probed by a comprehensive mutational study with a focus on single conserved color-tuning positions previously identified in other rhodopsins. None of the NeoR mutants have produced larger spectral shifts, especially when compared to the homolog substitution in other rhodopsins. For example, the highly conserved proline that resides close to the β-ionone ring in most microbial rhodopsins (see P186 in BR, Figure 4B) is replaced by threonine in NeoR and by isoleucine in Ruby-ACRs, which are the second most red-shifted microbial rhodopsins reported to date (λ_MAX_ 610 nm). Insertion of proline at this position has produced similar blue-shifts in both proteins [∼610 cm^−1^ NeoR (T238P); ∼440 cm^−1^ Ruby-ACR (I217P)] and, moreover, a corresponding red-shift has been observed for the reverse mutation in the sodium-pump KR2 [∼600 cm^−1^ KR2 (P219T), (Inoue et al., 2019)]. This demonstrates a similar impact of this particular residue on retinal absorption in all three proteins, which is irrespective of their different overall absorption. Remarkably, this proline is targeted in red-shifted rhodopsin mutants from directed evolution (McIsaac et al., 2014; Engqvist et al., 2015). Furthermore, blue-absorbing RGC2 (λ_MAX_ 480 nm) and green-absorbing RGC1 (λ_MAX_ 550 nm) from *R. globosum* also differ in this position. Other conserved residues close to the β-ionone ring, e.g., NeoR S191 (Figures 4A,D), again show similar blue-shifting [∼320 cm^−1^ NeoR (S191A)] capacity as observed in other microbial rhodopsins [∼240 cm^−1^ Gloeobacter Rhodopsin (S141A); ∼560 cm^−1^ BR (S141A) (Marti et al., 1991; Engqvist et al., 2015)]. Accordingly, the chromophore cavity of NeoR resembles a commonly observed impact for color-tuning and thus is not the structural feature that drives NIR-absorption of the protein. Notably, both mutations may lead to more flexibility of the retinal cavity, as indicated by spectral broadening (Figure 4D). Smaller red-shifts are obtained by mutations along the polyene chain (E141C, Y195, W234F; Figure 4A), which suggests that the absolute limit for NIR-absorption may not have been reached yet. Some residues of the NeoR retinal binding pocket cannot be studied experimentally since the mutant protein does not bind retinal. The position NeoR I237 attracts interest, which, as part of the highly conserved aromatic triad, is occupied by either phenylalanine or tyrosine in all other microbial rhodopsins. In the available structures, the aromatic ring aligns in parallel with the polyene chain of the retinal which suggests a strong electronic coupling. For Y185, the homolog in BR (Figure 4B), computational studies have indicated that there is a partial electron transfer towards the retinal in the excited state, which significantly contributes to the absorption energy and possibly influences excited state dynamics (Hayashi et al., 2012; Nass Kovacs et al., 2019). The only reported substitution, Y185F, alters the equilibrium of retinal isomers in dark-adapted BR and leads to a non-functional state that resembles red-shifted absorption of the O-photointermediate (Rath et al., 1993).

### Fluorescence Properties of Microbial Rhodopsins

**FIGURE 5:**
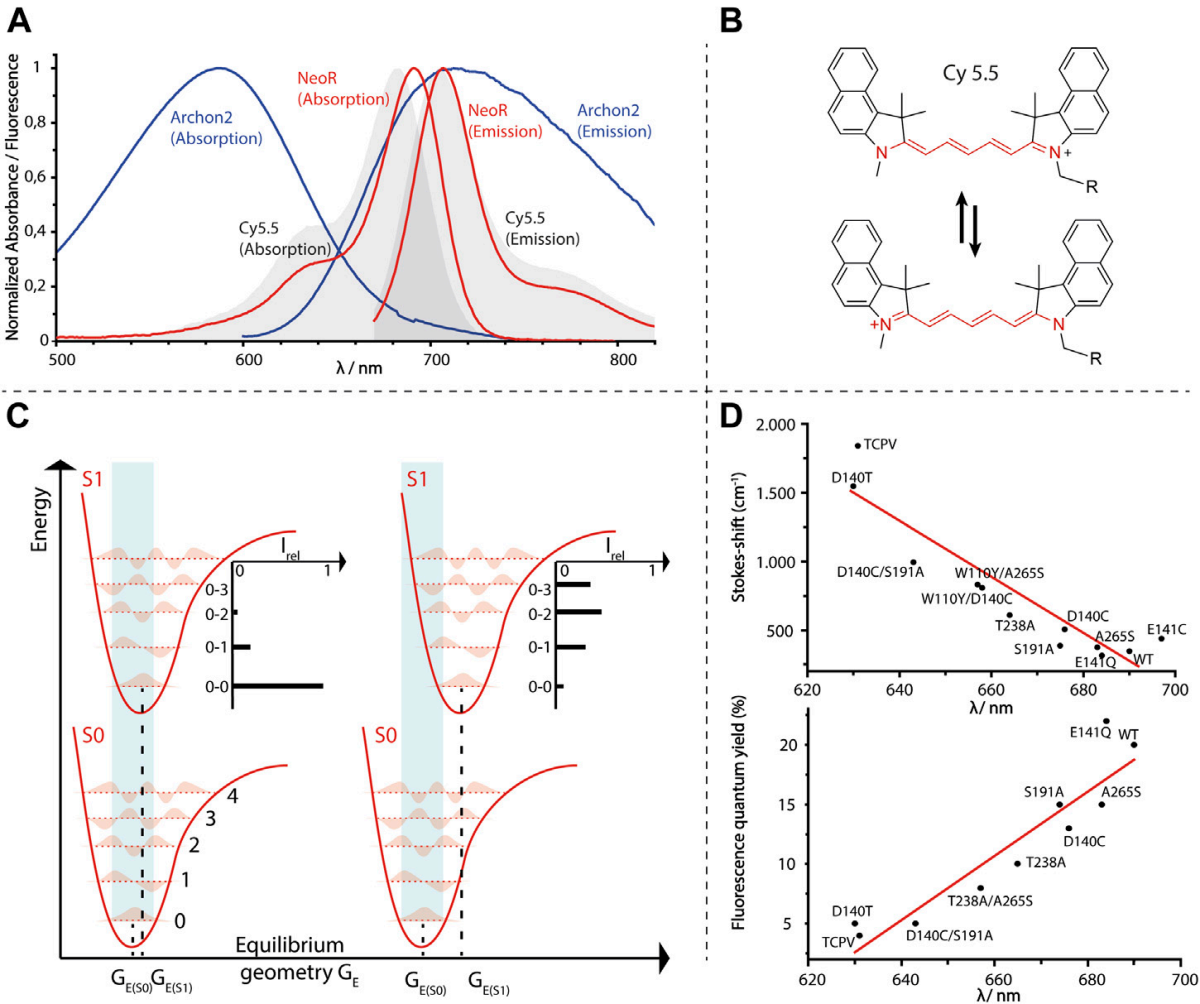
**(A)** Normalized absorption- and emission-spectra of Arch3-derived voltage sensor Archon2 [A. Silapetere (Humboldt University Berlin (Penzkofer et al., 2020)], NeoR and the fluorescence dye Cy 5.5. **(B)** Structure of symmetric cyanine dye Cy 5.5 with resonance formula representing the “cyanine-limit” of fully charge-delocalization (red). **(C)** Energy diagram of light-absorption (light-blue area) of a chromophore with either minor (left) or larger geometrical changes (right) upon excitation. Five vibronic-states for each electronic potential are shown. Different transition probabilities according to the Franck-Condon principle result an intensity-distribution regarding to vibronic-states of S1 as indicated. Note that the energy gap between S0 and S1 is the same for both examples. **(D)** Opsin-shifts of various NeoR mutants versus Stokes-shifts (cm^−1^, upper diagram) or fluorescence quantum yield [%, lower diagram, data taken from (Broser et al., 2020)].

The discovery that ChRs can be easily used to control membrane potentials, e.g., when expressed in neurons, has driven the demand for other optogenetic tools that allow an equally robust way to read out neuronal activity. Several approaches have been developed in order to design fluorescence probes that monitor changes in the membrane voltage (Panzera and Hoppa, 2019; Zhang et al., 2021). Among the genetically encoded voltage indicators (GEVI) developed so far, rhodopsin-based systems play a prominent role, as they respond highly sensitive and quickly to altered membrane potentials. A major limitation of these systems is their weak fluorescence, which results in low brightness and consequently the need for high light-intensities making them inapplicable for experiments with living rodents (Table 1). The low fluorescence of rhodopsins has been attributed to ultrafast processes such as retinal-isomerization occurring within the fs time regime after light excitation, that effectively depopulate the S1-state. Increased retinal fluorescence has been observed for states of BR and related proton-pump Archaerhodopsin-3 (Arch3, *Halorubrum sodomense*), which are derived from excitation of distinct photocycle intermediates (Ohtani et al., 1992; Maclaurin et al., 2013). As photoexcitation of these states rarely occurs under natural conditions, their structures have not been tuned for fast photoisomerization. Several attempts to increase the fluorescence quantum yield (φ_F_) of microbial rhodopsins have been undertaken. These have been mostly based on using Arch3 as the template (Hochbaum et al., 2014; McIsaac et al., 2014; Piatkevich et al., 2018) and have so far raised φ_F_ from ∼9 × 10^−4^ of wildtype Arch3 up to ∼1 × 10^−2^ [Archon2 (Piatkevich et al., 2018), Table 1], which has made the monitoring of multiple neuron activities in living mice feasible (Piatkevich et al., 2019). The employed mutations abolish a proton-pumping photocycle, increase the φ_F_ of the dark-adapted state, and have shown a broad, unstructured emission in the NIR-region (Figure 5A).

Regarding the difficulties to increase the retinal fluorescence in these systems, a further unexpected feature of NeoR is its strong fluorescence. The NeoR φ_F_ of 2 × 10^−1^ is more than one order of magnitude larger than any reported rhodopsin and even exceeds recently developed bilin-based fluorescent proteins [Table 1 (Oliinyk et al., 2019; Broser et al., 2020)]. Taking into account the high extinction coefficients (129,000 ^−M −cm^) due to the narrow absorption of NeoR, it is the brightest fluorescent protein with maximum emission above 700 nm (maximum emission 707 nm, Figure 5A and Table 1). According to the high fluorescence quantum yield, we have observed an extraordinary prolonged excited-state life-time of 1.1 ns for NeoR, which corresponds to a radiation lifetime of ∼5.5 ns as determined for other microbial rhodopsins [e.g., 6 ns in Proteorhodopsin, (Lenz et al., 2006)]. The resulted Stoke-shift of 17 nm (349 cm^−1^) has been found to be extraordinary small, and the absorption and emission spectra has shown pronounced mirror symmetry. Accordingly, the Franck-Condon region has been observed to be energetically close to the S1 excited state minimum, and thus only minor geometrical changes of the chromophore have occurred upon the S0 to S1 transition. Indeed, the spectral shape of NeoR absorption and emission closely resemble that of symmetric cyanine dyes (Figure 5A) in which the positive charge is almost completely delocalized along the polymethine chain (Figure 5B). Therefore, bond lengths alteration (BLA) in these molecules is determined to be low, and changes upon excitation are only minor (Eskandari et al., 2020). Consequently, the equilibrium geometry at the potential energy surfaces (PES) minima of the ground and excited state are nearby and favor the sharp spectral transition between the lowest vibronic states in S0 and S1 (0-0), which is characteristic for symmetric cyanine dyes (Mustroph et al., 2009) (Figure 5C). Based on the strong spectral similarity to cyanines regarding their narrow spectral-shape, the small Stokes-shift, and high fluorescence-quantum yield, one may postulate a similar charge-delocalization can be achieved for retinal in NeoR. The rigidity of the chromophore in NeoR has been clearly seen to increase the transition probability towards lower vibrational states in S1. Therefore, the maximum of absorption concentrates further red-shifted than in other systems in which the chromophore structure is more disturbed by the electronic transition (Figure 5C).

BLA is believed to play a prominent role in the photoisomerization of rhodopsins, as the weakening of the double bond character of C13 = C14 (or C11 = C12 in visual rhodopsins) in the excited S1-state facilitates a rotation around this bond (Gozem et al., 2017). This rotation is energetically not feasible in the ground state (Figure 3). Thus, extended charge delocalization and low BLA in NeoR are in conflict with efficient photo-isomerization. This explains the low quantum yield of NeoR photoproduct formation deduced from the high light-intensity required for photoconversion of the bistable rhodopsin. NeoR mutants that shift absorption to the blue have decreased fluorescence quantum yields and enlarged Stokes-shifts, which suggests a higher degree of geometrical relaxation of the respective excited chromophore structure (Figure 5D). The excited state dynamic for common photoisomerization from all-*trans* to 13-*cis* retinal in microbial rhodopsin can be described by a three-state, three modes model. According to this, progression of excited wave-packets from the Franck-Condon region follows a relaxation path accompanied by changes of BLA, torsion, and HOOP (hydrogen-out of plane)-vibrations to reach a conical intersection (CI), as given by the closest approximation of the PES of S1 and S0. While light-driven isomerization from 11-*cis* (in animal rhodopsins) or 13-cis (occurring in some microbial rhodopsins) to all-*trans* proceeds barrierless in S1 (Wand et al., 2011), photoisomerization starting from all-*trans* retinal has an energy barrier leaving the retinal in a tumbling state before reaching the CI (Kobayashi et al., 2001; Ernst et al., 2014). The origin of this barrier likely relies on the coupling between S1- and optical forbidden S2-state. Indeed, recent computational studies on Anabaena sensory rhodopsin (ASR) explain the increased retinal fluorescence of a mutant protein to result from PES degeneration by mixing of S1/S2, which provides or increases an activation barrier (Marín et al., 2019). This has been attributed to the small geometric effects of the retinal introduced in the strongly blue shifted mutant, rather than by the altered electrostatic interaction of the protein surrounding. Notably, the mutations that increase ASR fluorescence include the NeoR I237 homolog position discussed above, which suggests that multiple mechanisms may contribute to the strong fluorescence of NeoR. Clearly, the narrow NIR-absorption, low photoproduct quantum yield, and high fluorescence of NeoR suggest that the structure of PES involved in the photochemical reactions are significantly different to conventional rhodopsins. Studying the dynamics of the excited state of NeoR by ultrafast spectroscopy would therefore be an important approach to understand the source of its high fluorescence.

### Color-Tuned Retinal-Analogs in Microbial Rhodopsins

Numerous attempts have been made to incorporate chromophores other than all-*trans* retinal into the binding pocket of microbial rhodopsins to either study the photochemistry of the photoreceptor or to obtain red-shifted and/or highly fluorescent variants, e.g., as potential optogenetic tools (Derguini et al., 1983; Asato et al., 2002; Gaertner et al., 2002; Ganapathy et al., 2017; Herwig et al., 2017). Beside the naturally occurring 3,4 dihydro-retinal (A2) with red-shifted absorption due to an additional conjugal C=C double bond at the β-ionone ring, several artificially synthetized chromophores with methyl-substitution, extended π-systems, cyanine-structure, or locked-chromophores have been successfully incorporated in proton-pumping rhodopsins from archaea or eubacteria. Reconstitution with A2-retinal has led to red-shifted absorption of around ∼22–32 nm (∼680–1,100 cm^−1^) for common green/yellow-absorbing rhodopsins (Sineshchekov et al., 2012), while A2-retinal in NeoR has revealed a further red-shift by 69 nm [Figure 1 (Broser et al., 2020)]. Even when considering that this shift corresponds to only ∼1,300 cm^−1^ due to the low energy-range of NeoR-absorption, it is still significantly higher than what has been observed for other rhodopsins. Highly red-shifted variants of BR (even above 700 nm) with narrow absorption spectra have been obtained using Azulenic-based chromophores or Merocyanines, the latter forming nearly symmetric cyanines upon opsin-binding (Derguini et al., 1983; Asato et al., 2002). A recently optimized Arch3 mutant with cyanine-Schiff base chromophore has exhibited narrow band absorption and fluorescence in the NIR with a fluorescence quantum yield φ_F_ of 1.3 × 10^−1^ [Table 1 (80)]. However, even in solution, the optical properties of these analogs substantially differ from that of all-*trans* retinal to the point that the spectral tuning caused by the binding of the chromophore to the opsin has been found to be essentially similar to the native protonated RSBH^+^. In contrast, NIR-absorption and high fluorescence has been attributed to the natural retinal chromophore in NeoR, rendering its protein-induced opsin-shift as being much higher than in these analog reconstituted rhodopsins. Despite their potential in terms of color tuning and fluorescence enhancement, a major drawback of using retinal analogs in optogenetics and fluorescence imaging is that the synthetized chromophore must be delivered externally and competes for the endogenous retinal that is present in most cells.

### Heterodimeric Structure of RGCs From *R. globosum*

**FIGURE 6:**
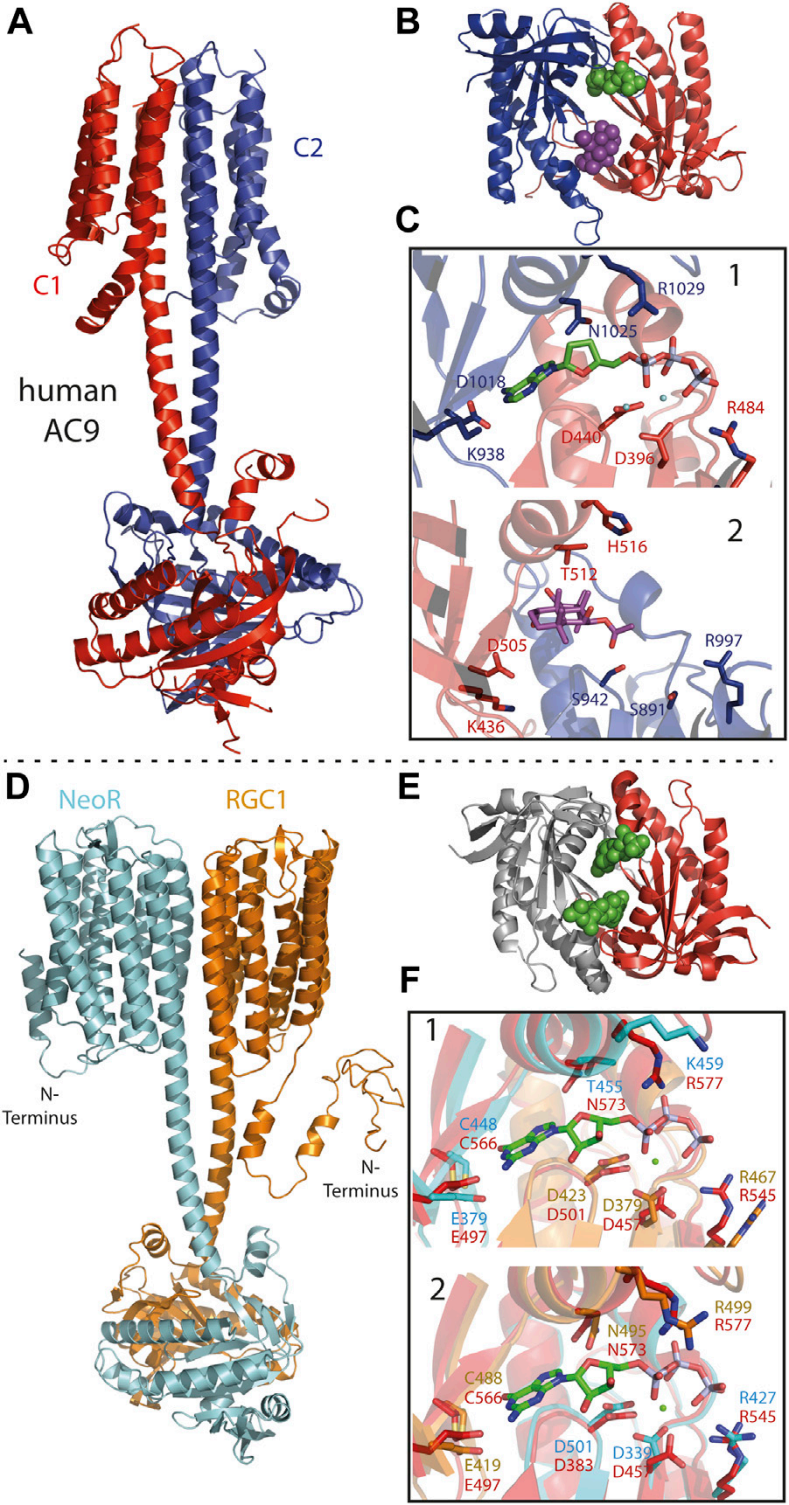
**(A)** Cryo-EM structure of pseudo-heterodimeric human membrane adenylyl cyclase 9 [AC9; 6R4O (Qi et al., 2019)] with C1- and C2-subunits colored red and blue. **(B)** Crystal-structure of pseudo-heterodimeric AC chimera [C1a/C2a: VC1(dog)/IIC1(rat) colored as in c; 1CJU (Tesmer et al., 1999)] with substrate analog (2′,3′-Dideoxy-ATP) as green and forskolin as violet spheres. **(C)** Close-up of the pseudosymmetric active site: 1) catalytic-site with adenine bound to C2a (blue) and phosphate to C1a (red) subunit. Catalytic relevant residues depicted as sticks. 2) Regulatory, forskolin-binding site, residues homolog to 1 shown. Despite the different models used in A and B the general architecture of the sites 1 and 2 remains similar. **(D)** Structural model of the NeoR/RGC1 heterodimeric complex as derived from Alphafold2 (Mirdita et al., 2021), conducted by E. Peter (Humboldt University Berlin). Color-code: NeoR (cyan) and RGC1 (orange). **(E)** Crystal-structure of the cyclase-core of homodimer RGC from *C. anguillulae* [6SIR (Butryn et al., 2020)] GTP substrate drawn as green spheres indicating the two symmetric catalytic sites at the dimerization interface. **(F)** Structural overlay of the NeoR/RGC1 pseudosymmetric catalytic sites (colored as in D) to the homodimer from *C. anguillulae* (red) with bound GTP and Ca^2+^ (green spheres). Site 1: guanine-base bound to NeoR, phosphate bound to RGC1; Site 2 with opposite contributions.

For the many microbial rhodopsins with different functionalities, a variety of homo-oligomeric forms have been reported (Shibata et al., 2018), but their functional relevance, apart from protein stability and packing, is still not fully understood. For Enzymerhodopsins, oligomerization is determined by the dimeric structure of the subsequent functional enzyme-domains. With some exceptions, the vast majority of histidine-kinases and phosphodiesterases function as homodimers, while class III cyclases form either functional homo- or heterodimers (Steegborn, 2014). *B. emersonii* and the related fungus *Catenaria anguillulae,* carry only a single RGC gene, so that the RGCs initially discovered in these organisms function as homodimers. The homodimeric cyclase-core of RGC from *C. anguillulae* arranges similar to other class III cyclases yielding two symmetrically catalytic sites located at the dimerization interface [Figure 6 (Scheib et al., 2018; Butryn et al., 2020)]. Both monomers provided catalytic relevant residues, which are complementary involved in nucleotide-binding and catalytic turnover. The three RGCs from *R. globosum* are obligate heterodimers and light-triggered cyclase activity is elusively present for co-expression of NeoR with either RGC1 or RGC2 (Broser et al., 2020). Nevertheless, the cyclase domains of RGC1 and RGC2 have ∼60% sequence identity to homodimeric RGCs which includes all residues known to be relevant for cyclization (Figure 6F). This differs from many known heterodimeric class III cyclases, where the absence of catalytic residues requires heterodimerization to complement the catalytic active-site for enzymatic functioning (e.g., tmAC as shown in Figure 6C). Thus, the structural reason for the obligate heterodimerization of the photoreceptors in *R. globosum* probably lies in the less conserved rhodopsin domains or linker sequences. Enzymerhodopsins, particularly RGC sequences, have a prolonged N-terminus preceding the additional transmembrane α-helix (Figures 2A, 6D). For homodimeric RGCs, the cytoplasmic N-terminus is involved in enzyme activation (Fischer et al., 2021) and impaired light-regulation with high-dark activity was observed upon its truncation (Gao et al., 2015). Remarkably, all three RGCs of *R. globosum* have significantly shorter N-termini than homodimeric RGCs, and future experiments may explore the impact of this part on functional heterodimerization.

In particular, class III cyclases of higher eukaryotes are known to function as heterodimeric complexes, with the cyclase domains forming two pseudosymmetric active-sites of different functionality. For example, in mammalian membrane-bound adenylyl-cyclase (tmAC), a pseudo-heterodimer coded within one polypeptide chain, binding of the diterpene forskolin to the degenerated non-catalytic site of the cyclase core strongly increase the enzyme-activity of the remaining active-site (Figures 6B,C) (Steegborn, 2014; Qi et al., 2019). The structural model of the RGC1/NeoR heterodimer, as obtained from alphafold2, has shown a remarkably similar overall arrangement to the cryo-EM structure of human tmAC9 (Figures 6A,D) (Qi et al., 2019; Mirdita et al., 2021). In both models, the membrane-domain is connected to the cyclase by a ∼5 nm α-helical coiled-coil linker that extends the two α-transmembrane helices located at the dimerization interface of the membrane subunits. In contrast to rhodopsins, hetero-oligomeric complexes have been described for several types of seven transmembrane α-helix receptors, such as the insect odorant receptors and G-protein coupled receptors (Neuhaus et al., 2005; Shen et al., 2021; Wicher and Miazzi, 2021). Apparently, hetero-oligomerization enables an additional degree of regulation in all of these complexes, and the mechanism of how each subunit contributes to signal-transduction is a topic of current research. For RGC2/NeoR heterodimers, blue-light evoked photocurrent has been observed to increase moderately (∼1.4x) after the bistable NIR-chromophore in NeoR was photoconverted to its UV-absorbing state (Broser et al., 2020). While this indicates a regulatory function of the NIR-photoreceptor within the heterodimer, the mechanism of how NeoR influences light-triggered enzyme activation is currently unknown. The observed function is in accordance with the low quantum yield of NeoR photoconversion, which precludes the efficient and fast photoresponse to a light-stimulus. As a bistable photochromic photoreceptor, NeoR is capable of accumulating photons over an extended period of time, thereby adapting the heterodimer to slowly changing environmental light conditions. In its mode of action, NeoR resembles NIR-sensitive phytochromes, which also show low efficient photoconversion (Consiglieri et al., 2019).

### Outlook

The impact of optogenetics and the availability of various genomic data have led to a renaissance of microbial rhodopsins in scientific research over the past decade. In particular, the use of ChRs as neuronal modulators in biological tissues, including whole animals, or the prospective of therapeutic applications in humans has encouraged researchers to trim the absorption of this photoreceptor class to the NIR region of maximum transparency. However, the outcome of most color-tuning approaches has been exceeded by the discovery of further red-shifted natural variants, such as Chrimson or recently discovered Ruby-ACRs [λ_MAX_ 590 nm–610 nm, (Govorunova et al., 2020)]. Taking the absorption of Sensory rhodopsin I (SRI, λ_MAX_ 587 nm, Table 1), which have been already known about for 4 decades (Bogomolni and Spudich, 1982), the achieved extension regarding the red spectral-limit of rhodopsin is rather low. In this regard, the discovery of NIR-absorbing retinal-chromophore in NeoR marks a substantial step toward our understanding of the spectral diversity covered by rhodopsins. Given the limited number of Enzymerhodopsins characterized to date and the tremendous progress in the identification of novel rhodopsins from across the microbial world, one can speculate about further members of this photoreceptor class occupying the far-red spectral region. Indeed, similar heterodimeric RGCs have been found in the genome of the closely related fungus *Chytridium confervae* (van de Vossenberg et al., 2019), including a bistable NeoR variant absorbing in the NIR-range (λ_MAX_ 677 nm). This indicates a more widely distribution of heterodimeric RGCs in certain fungi (Broser et al., 2020).

From what we currently know about NeoR, it is the special spatial arrangement of the counterion complex that allows for NIR absorption. Still, it is puzzling how putative cyanine-like charge delocalization is achieved and future spectroscopic and structural investigations are essential for a more detailed understanding. Along with this, it is difficult to assume the absolute low-energy limit of retinal chromophores. The effects of all nine blue-shifting single mutants obtained from individual residues of the NeoR retinal cavity (excluding counterion mutants) add to 0.24 eV, which agrees surprisingly well with the spectral impact assigned to the binding pocket in SR II (Hoffmann et al., 2006). This contribution would shift the absorption maximum of NeoR to ∼610 nm, resembling RSBH^+^ *in vacuo*. However, already three mutants [E141C, W195F and W234F (Broser et al., 2020)] have been found to absorb further red than wildtype NeoR, suggesting some potential for variants with maximal sensitivity above 700 nm. The rigid chromophore constellation that excludes water from RSBH^+^ and restricts photoisomerization might limit the potential of active triggering processes. Therefore, NeoR-like retinal chromophores in nature may be confined to Enzymerhodopsins and other light sensors that, unlike ion-transporting pumps or channels, do not rely on high efficiency and the access of water to the RSBH^+^ active site. Nevertheless, the retinal-binding pocket in NeoR is still remarkably similar to other microbial rhodopsins, which suggests that far-red absorption and high fluorescence could be adapted to some level in other proteins. This may be particularly relevant for the development of voltage sensors operating at lower light intensities in the far-red. While the photocurrents that have been observed in conjunction with RGC2/NeoR heterodimers expressed in mammalian cells suggest some plasma-membrane localization, as of yet we have not been able to detect any membrane voltage dependent changes in NeoR fluorescence (Broser et al., 2020). For this purpose, a more detailed understanding of the NeoR chromophore structure and photochemistry combined with directed evolution methods may guide future NeoR-based sensor applications. Still, due to the abundance of retinal in most biological tissue, NeoR holds great potential as bright fluorescence marker, which can be excited by NIR or above when employing multi-photon excitation. Another striking novel feature is heterodimerization of functional RGC/NeoR photoreceptors. Thus, these complexes are comprised of two distinct chromophores with different functionality. In fact, the term Neorhodopsin alludes to Neochromes, a photoreceptor class that also contains two different photoactive pigments. The structural features that determine heterodimerization, as well as the interaction of the two chromophores and the mechanism of how NeoR modulates blue/green light-triggered enzyme activation remain to be identified. Many microbes contain multiple opsin genes, such as the several different HKR sequences found in various species (Kateriya et al., 2004; Awasthi et al., 2020), which suggests that other microbial rhodopsins may also form heterocomplexes. In this respect, the discovery of heterodimeric RGC may lead to a new understanding of how these ancient photosystems are functioning.
